# Combinations of BRAF inhibitor and anti-PD-1/PD-L1 antibody improve survival and tumour immunity in an immunocompetent model of orthotopic murine anaplastic thyroid cancer

**DOI:** 10.1038/s41416-018-0296-2

**Published:** 2018-10-17

**Authors:** Viswanath Gunda, Benjamin Gigliotti, Dorothy Ndishabandi, Tameem Ashry, Michael McCarthy, Zhiheng Zhou, Salma Amin, Gordon J. Freeman, Alessandro Alessandrini, Sareh Parangi

**Affiliations:** 1 Department of Surgery, Massachusetts General Hospital, Harvard Medical School, Boston, MA USA; 2 Department of Medicine, Massachusetts General Hospital, Harvard Medical School, Boston, MA USA; 3 Department of Medical Oncology, Dana-Farber Cancer Institute, Harvard Medical School, Boston, MA USA

**Keywords:** Head and neck cancer, Preclinical research, Tumour immunology, Targeted therapies, Cancer models

## Abstract

**Background:**

Patients with anaplastic thyroid cancer (ATC) have an extremely poor prognosis despite aggressive multimodal therapy. ATC has a high prevalence of *BRAF*^*V600E*^ mutations and is associated with an immunosuppressive microenvironment; we previously demonstrated that the combination of BRAF inhibitor and checkpoint inhibitor immunotherapy synergistically reduce tumour volume in an immunocompetent mouse model of orthotopic ATC.

**Methods:**

We again utilised our mouse model of ATC to assess the combination of BRAF^V600E^ inhibitor PLX4720 and anti-PD-L1 or anti-PD-1 antibody on survival, and performed immune cell profiling of lymphoid and myeloid-lineage cells during maximal treatment response and tumour regrowth.

**Results:**

Combination therapy dramatically improved mouse survival. Maximal tumour reduction was associated with increases in the number and cytotoxicity of CD8^+^ T cells and NK cells, as well as increases in mostly M1-polarised tumour-associated macrophages (TAM) and decreases in myeloid-derived suppressor-like cells. Regrowth of tumour occurred after 2–3 weeks of ongoing combination therapy, and was most significantly associated with decreased TAMs and a dramatic increase in M2-polarisation.

**Conclusions:**

Combination of PLX4720 and anti-PD-L1/PD-1 antibody dramatically reduced tumour volume, prolonged survival and improved the anti-tumour immune profile in murine ATC. Tumour growth inevitably recurred and demonstrated re-emergence of an immunosuppressive tumour microenvironment.

## Background

The incidence of thyroid cancer is steadily increasing worldwide.^1^ The majority of patients have well differentiated thyroid cancers (DTC) that respond well to total thyroidectomy, and if needed, radioactive iodine and thyroid hormone suppression.^2^ In contrast, anaplastic thyroid cancer (ATC) is an extremely rare and aggressive tumour that may arise de novo or from a pre-existing DTC; patients almost invariably die from their disease despite aggressive treatment.^3,4^ Although the best treatment strategy remains unclear, patients are typically treated with multimodal therapy including surgery, combination chemotherapy, and external beam radiation. Unfortunately, despite refinement of each component of multimodal therapy, the prognosis remains dismal in stage IVc and recurrent disease; new systemic therapies are urgently needed.^5^

ATC has been increasingly recognised to harbour complex genomic changes and instability, including genome copy number alterations, gene fusions, and mutations in *TP53*, *BRAF*, *NRAS*, the *TERT* promoter, as well as genes crucial for epigenetic regulation.^6,7^ Given the high frequency of *BRAF*^*V600E*^ mutations in ATC, selective BRAF^V600E^ inhibition (BRAFi) is an attractive option to improve treatment specificity and minimise toxicity, and has quickly become standard of care in other *BRAF*^*V600E*^-mutant malignancies such as melanoma.^8^ However, despite initial impressive response rates, the durability of response is limited owing to development of resistance.^9–11^ Since reactivation of downstream MEK is a frequent mechanism of resistance, MEK inhibition (MEKi) is a useful adjunct; the combination of BRAFi (dabrafenib) and MEKi (trametinib) demonstrated an overall response rate of 69% in a recent phase II clinical trial which has led to FDA approval of the combination.^12^

In addition to tumour-intrinsic factors, an immunosuppressive microenvironment has been recognised as a contributor to thyroid cancer growth and metastasis.^13,14^ Immune cells such as tumour-associated macrophages (TAMs), myeloid-derived suppressor cells (MDSCs), and regulatory T cells (Tregs) have been shown to inhibit anti-tumour T cell function. Our group and others have shown that *BRAF*^*V600E*^-mutant thyroid cancers have increased expression of Programmed Death Ligand 1 (PD-L1, CD274), a physiologic “checkpoint” expressed on tumour cells, macrophages, and other cell types. PD-L1 binds to its receptor, PD-1, on effector immune cells, such as T cells, and inhibits proliferation, cytotoxicity, and cytokine production. The development of neutralizing monoclonal antibodies to PD-1/PD-L1 (along with other critical immune checkpoints, such as CTLA-4) has led to improved patient survival in many cancer types.^15,16^ Initial reports of the use of anti-PD-1 in thyroid cancer (pembrolizumab in metastatic DTC, spartalizumab in ATC) show promise, although response rates are variable.^17,18^

Combinatorial strategies that include agents with multiple mechanisms of action are a potential means to improve efficacy and forestall the development of resistance.^13,19^ The addition of immunotherapy to agents directed against angiogenesis, proliferation, oncogenic mutations, etc. has recently seen an accelerated progression from bench to clinical trials, and there is great interest in using actionable genetic information from tumour/cell-free DNA to personalise selection of targeted therapies.^10,20^ However, given the unique challenge of enroling patients with such a rapidly progressive disease, mouse models have become an invaluable tool to advance our understanding of the biology of ATC, to trial new drugs and combination regimens, and to investigate predictors of response and mechanisms of resistance. As these mechanisms are elucidated, logical combinations will be easier to propose and investigate.^12,21–23^

Given the prevalence of *BRAF*^*V600E*^ mutations in ATC and its association with higher PD-L1 expression and increased numbers of tumour-infiltrating T cells, the combination of *BRAF*^*V600E*^ inhibitors with immunotherapy is of particular interest.^24,25^ Our previous study demonstrated that MAPK pathway activity modulates thyroid tumour PD-L1 expression, and that the combination of PLX4720 and anti-PD-L1 antibody in an immunocompetent murine model of orthotopic ATC led to a superior and synergistic anti-tumour response compared to either monotherapy alone; tumour shrinkage was associated with increases in CD8^+^ T cells and granzyme B staining.^24^ These preliminary data informed development of an ATC-specific clinical trial to examine the safety and efficacy of atezolizumab with various targeted therapies (cobimetinib, vemurafenib, and bevacizumab) or paclitaxel (NCT03181100) based on the mutation status of the tumour.

In this follow-up study, we sought to assess the effect of combination therapy on survival, and expanded our treatment with anti-PD-1 antibody, which has seen wide clinical use. Since our model is extremely aggressive and regrowth of ATC is essentially inevitable in humans, we extended our experiments to assess for tumour regrowth and to more thoroughly characterise the tumour immune signature of both lymphoid and myeloid lineage cells. We hypothesised that the combination of BRAF inhibition and anti-PD-1/PD-L1 antibody therapy would improve survival,  that tumour regression would be associated with improved immune-mediated anti-tumour cytotoxicity, and that tumour would eventually recur and show re-emergence of an ineffective immune response. To our knowledge, this is the first study to show improved survival with *BRAF*^*V600E*^ inhibitor and anti-PD-1/PD-L1 combination therapy, and to conduct immune profiling during maximal treatment response and after regrowth of the tumour.

## Materials and methods

### Cell culture, reagents, and antibodies

One murine thyroid cancer cell line, TBP-3743, was used in the study.^26^ The cell line cultured in Dulbecco’s Modified Eagle’s Medium (DMEM) supplemented with 10% foetal bovine serum and penicillin/streptomycin and incubated at 37 °C in a 5% CO_2_ incubator.

The anti-PD-L1 (10F.9G2) and anti-PD-1 (332.8H3) antibodies have been previously characterised; both had less than 2EU endotoxin per mg protein and were injected intraperitoneally (I.P.), as described below.^27^ LEAF™ Purified anti-mouse NK1.1 (PK136, BioLegend) antibody was used for NK cell depletion, as previously described.^28^

For flow cytometry, anti-F4/80-FITC (Isotype rat IgG2a,k), and anti-Ly6C-PerCP/cy5.5 (Isotype rat IgG2c,k) antibodies were purchased from eBioscience (San Diego, CA). Anti-Ly6G-PE (Isotype rat IgG2a,k), anti-CD206^−^PE (Isotype rat IgG2a,k), anti-IA/IE-PerCP/cy5.5 (Isotype rate IgG2b,k), anti-CD274-PerCP/cy5.5 (Isotype rat IgG2b,k), anti-NK1.1-PE/cy7 (Isotype rat IgG2a,k), and anti-CD11b-APC/cy7 (Isotype rat IgG2b,k) antibodies were purchased from BioLegend (San Diego, CA).

### RNA isolation and real-time polymerase chain reaction

RNA isolation was performed using Trizol (Invitrogen, Carlsbad, CA) and cDNA was synthesised using the Superscript VILO cDNA synthesis kit (Invitrogen), according to the manufacturer’s instructions. PD-L1 (Primer ID Hs-01125301_m1, Mm00452054_m1) and H2-K^b^ gene expression (F-GCTGGTGAAGCAGAGAGACTCAG, R-GGTGACTTTATCTTCAGGTCTGCT) were measured using TaqMan Gene Expression Assays (Invitrogen) by real-time reverse transcriptase polymerase chain reaction (RT–PCR) with technical triplicates and two repetitions. Gene expression levels were normalised to GAPDH expression.^29^

### Immunocompetent orthotopic murine model of ATC

**Fig. 1:**
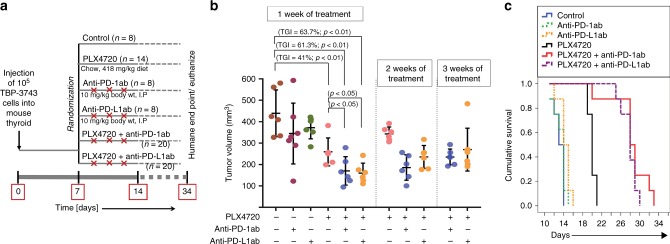
Treatment of immunocompetent mice with orthotopic ATC (TBP-3743) with PLX4720 and/or anti-PD-1/anti-PD-L1 as single agents and combination therapy; experiment was repeated twice. **a** Experimental design, treatment randomisation, and schedule. **b** Combination therapy for 1 week led to 61.3% (PLX4720 + anti-PD-1) and 63.7% (PLX4720 + anti-PD-L1) tumour growth inhibition vs. controls (*p* < 0.01). **c** Combination-treated mice survived for a median of 28 ± 2.5 days (PLX4720 + anti-PD-1) and 28 ± 0.9 days (PLX4720 + anti-PD-L1), both significantly longer than controls (*p* < 0.001)

Seventy-eight 6-week-old female B6129SF1/J mice were implanted with 10^5^ cells from the genetically engineered TBP (***T****POCreER*; ***B****raf* ^*tm1Mmcm/WZ*^; *Tr****p****53*^*tm1Brn/tm1Brn*^) 3743 BRAF^*V600E/WT*^ P53^*−/−*^ murine tumour cell line, as previously described.^26^ Briefly, mice were anesthetised, the thyroid gland was exposed, and 10^5^ cells were injected into the left lobe using a 27-gauge needle. On post-injection day 7, mice were randomised (see schema in Fig. 1a): the control group (*n* = 8, one timepoint at 1 week) received a control diet (Research Diets, Inc.), the PLX4270 group (*n* = 14, 7 for each timepoint at 1 week and 2 weeks) received PLX4720-impregnated chow (417 mg/kg body weight, Research Diets, Inc.) ad libitum, the anti-PD-1 and anti-PD-L1 groups (*n* = 8 each group, one timepoint at 1 week) received control diet and were injected with anti-PD-L1 or anti-PD-1 antibody at 200 µg/mouse I.P. twice weekly, and the combined treatment group received both PLX4720 chow and either anti-PD-L1 or anti-PD-1 antibody (*n* = 20 each group, 6–7 each timepoint at 1 week, 2 weeks, and 3 weeks). The mice were sacrificed at 2-weeks, 3-weeks, and 4-weeks post-implantation. Both fresh and formalin-fixed tumours were collected for RNA isolation and IHC analysis, respectively. Tumour volume was calculated using the formula (π/6) × length × width × height.^30^ The experiment was repeated twice. In order to generate a survival curve, the experiment was repeated using 48 (*n* = 8 per group) mice and utilizing humane endpoints (>20% weight loss, poor body condition score, poor grooming, inability to ambulate, etc. per IACUC protocol) rather than timed sacrifice.

### Immunohistochemistry

Formalin-fixed orthotopic thyroid tumours were cut into 4-μm sections and stained with Haematoxylin and Eosin. IHC analysis was performed for CD8 (Leica Microsystems), Granzyme B (abcam), and F4/80 (eBioscience). The primary antibody was detected using the respective biotin-free secondary antibody. Granzyme B staining was quantified using the mean percentage of total nucleated cells, averaged over five representative high-powered fields.

### Flow cytometry

Representative tumours were dissected and enzymatically digested with collagenase (100 μ/ml) and dispase (0.5 mg/ml) at 37 °C for 30–40 min, in preparation for flow cytometric analysis. Cells were washed twice with PBS, and fixed with 2% paraformaldehyde for 10 min on ice. Cells were again washed and then pre-incubated with Trustain fcX™ (anti-mouse CD16/32, BioLegend). Samples were stained with the primary antibodies listed above for 30 min, washed twice, and were resuspended in standard flow cytometric buffer. Cells were analysed using the BD FACSVerse™ flow cytometer (BD Biosciences, San Jose, CA). The data were processed using Flowjo software (v8.8, Tree Star, Inc., Ashland, OR).

### Milliplex® multiplex cytokine analysis

The Milliplex® MAP kit (MCYTOMAG-70K, EMD Millipore Corporation, Billerica, MA) was used according to manufacturer specifications. The specific analysed cytokines are listed in Fig. 3b.

### Statistical analysis

Statistical analysis was carried out using Microsoft Excel (Microsoft, Redmond, WA, USA), IBM SPSS Statistics (version 23.0, IBM, Armonk, NY), and GraphPad Prism 7 (Version 7.00, GraphPad Software, Inc., La Jolla, CA). All experiments were performed in technical triplicate and repeated twice unless specified otherwise. A fold change in mRNA expression of 2 or more was considered significant. A log power of 2 for fold changes in expression was used to generate box-and-whisker plots based on the median and interquartile range, and the non-parametric Mann–Whitney *U*-test was used to compare independent groups. Two-way factorial analysis of variance (ANOVA) was applied to test the effects of treatments on reduction in tumour volume. Two-tailed values of *P* < 0.05 were considered significant for all analyses. Kaplan–Meier survival analysis was performed to calculate fractional survival using the Logrank test for each group, compared against the control mice and monotherapy as reference; median survival was reported in days.

## Results

### Combination therapy with PLX4720 and anti-PD-1/PD-L1 antibody reduces tumour volume and leads to improved survival in an immunocompetent orthotopic murine model of anaplastic thyroid cancer

**Fig. 2:**
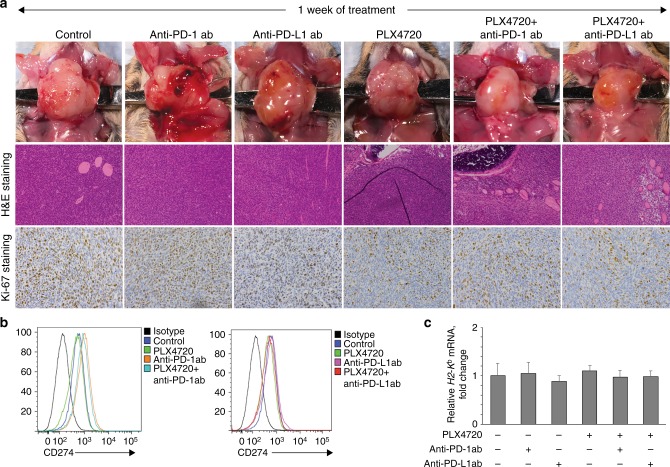
**a** Pictures of representative tumours after 1 week of treatment (upper), H&E staining (middle), and Ki67 staining (lower). All mice treated with PLX4720 singly or in combination had reduced cellular proliferation. **b** PD-L1 surface expression increased in all mice treated with anti-PD-1 singly or in combination. **c** None of the treatments led to changes in surface MHC-I expression. *N* = 4 mice per group, repeated twice

We utilised our previously described immunocompetent murine model of orthotopic ATC in which TBP-3743 cells were injected into the thyroid of female syngeneic mice (*n* = 78). One week after tumour implantation, when tumours were ~100 mm^3^, mice were randomised to receive either single drug (PLX4720, anti-PD-1, or anti-PD-L1), or combination treatment (see schema in Fig. 1a). After 1 week of treatment, as expected, all control animals met humane endpoints and were sacrificed; median tumour volume was 440 ± 109 mm^3^. A cohort of mice from each treatment group was also sacrificed for comparison. Treatment with PLX4720 led to significant tumour volume reduction (259 ± 65 mm^3^, 41% tumour growth inhibition, *p* < 0.01), whereas dramatic tumour reduction was seen with PLX4720 + anti-PD-1 (160 ± 47 mm^3^, 61% tumour growth inhibition, *p* < 0.01) and PLX4720 + anti-PD-L1 (170 ± 67 mm^3^, 64% tumour growth inhibition, *p* < 0.01), consistent with our prior work (Fig. 1b). Treatment with either anti-PD-1 or anti-PD-L1 antibody alone was ineffective. Pictures of representative tumours after 1 week of treatment are shown in Fig. 2a.

To investigate the effect of each treatment on survival, we repeated the experiment using predetermined physical evidence of animal pain and distress as endpoints, rather than time (*n* = 8 per group). Control mice survived for a median of 13 ± 0.5 days; anti-PD-1 and anti-PD-L1 groups did not survive for significantly longer. PLX4720-treated mice survived significantly longer than control, (median 20 ± 0.3 days, *p* < 0.001), whereas PLX4720 + anti-PD-1 (median 28 ± 2.5 days, *p* < 0.001) and PLX4720 + anti-PD-L1 (median 28 ± 0.9 days, *p* < 0.001) groups survived dramatically longer than control, and significantly longer than the PLX4720-treated group (*p* < 0.001) (Fig. 1c).

Tumour regrowth during PLX4720 emerged between 1 and 2 weeks of treatment; most mice died shortly after 2 weeks of treatment and had an average tumour volume of 343 ± 32 mm^3^. Regrowth during PLX4720 + anti-PD-1/PD-L1 combination therapy appeared between 2 and 3 weeks of treatment; most mice died shortly after 3 weeks of treatment. Tumour volume of PLX4720 + anti-PD-1-treated mice was 186 ± 59 at week 2, and 235 ± 41 at week 3; tumour size of PLX4720 + anti-PD-L1-treated mice was 235 ± 53 at week 2, and 270 ± 109 at week 3 (Fig. 1b). Mouse serum from each group was pooled and analysed for toxicity after 2 weeks of treatment (Supplemental Fig. 1).

### Combination therapy is associated with an anti-tumour immune signature

#### Cellular proliferation, PD-L1 expression, and MHC-I expression

Staining with anti-Ki-67 demonstrated a high degree of cellular proliferation in control, anti-PD-1, and anti-PD-L1-treated mice, with a marked decrease in proliferation for PLX4720-containing groups, confirming PLX4720 anti-tumour effect.^31^ Flow cytometry with anti-PD-L1 antibody showed increased PD-L1 surface expression in anti-PD-1-treated groups (Fig. 2b). Quantitative RT–PCR for H2-K^b^ mRNA, the MHC-I haplotype present in B6129SF1/J mice, showed no significant difference between treatment groups after 1 week of treatment (Fig. 2c), or at subsequent weeks (data not shown), confirming the immune profile changes were not due to alterations in MHC expression, which is a potent stimulus for NK/NKT cell-mediated destruction.^32^

#### Lymphoid-lineage cells: cytotoxic T cells, T-regulatory cells (Tregs) and NK cells

**Fig. 3:**
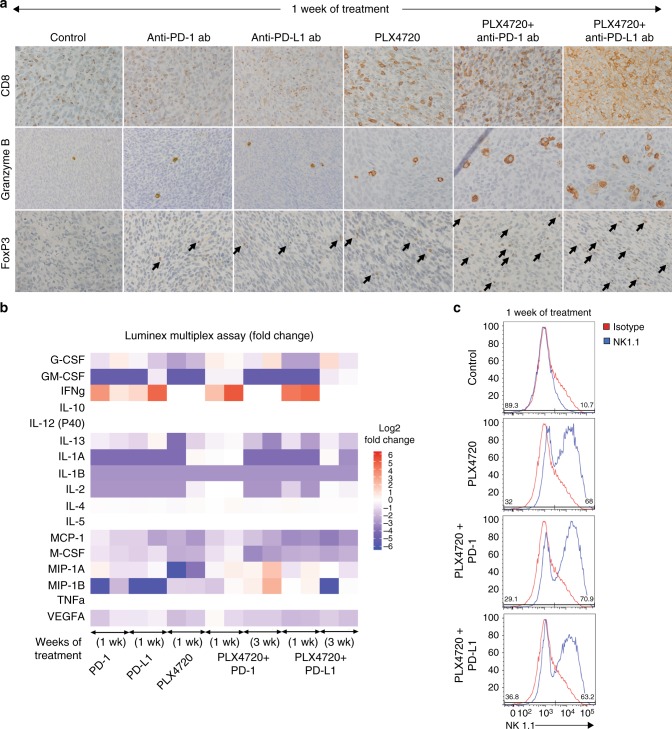
**a** One week of combination therapy led to a dramatic increase in CD8^+^ cytotoxic T cells, granzyme B staining, and FoxP3^+^ T cells. **b** Milliplex cytokine analysis showed complex changes after treatment for 1 and 3 weeks; IFNγ increased during maximal response and decreased during tumour regrowth compared to control. **c** All mice treated with PLX4720 singly or in combination had a dramatic increase NK cells (representative samples shown using NK1.1 antibody). *N* = 4 mice per group, repeated twice

Since tumour-infiltrating lymphocytes are the main effectors of immune-cell mediated death, immunohistochemistry was performed for the CD8^+^ T cell subset, granzyme B, a serine protease and marker of cytotoxicity of lymphocytes, and FoxP3, a putative marker of Tregs. (Fig. 3a) CD8^+^ staining was increased in anti-PD-1 and anti-PD-L1-treated groups but was much more significantly increased in PLX7420-treated mice, and most dramatically increased in combination-treated groups. Granzyme B staining was significantly higher in PLX4720 (1.33 ± 0.36%, *p* < 0.01), anti-PD-L1 (0.95 ± 0.17%; *p* < 0.01), PLX4720 + anti-PD-1 (2.28 ± 0.40%, *p* < 0.01), and PLX4720 + anti-PD-L1-treated groups (2.81 ± 0.78%, *p* < 0.01), compared to control (0.38 ± 0.13%). Mice treated with anti-PD-1 alone did not show increased Granzyme B staining, compared to control. FoxP3^+^ T cells modestly increased with all three monotherapies, but dramatically increased with both combination therapies.

Flow cytometry with anti-NK1.1, an NK cell (and NKT cell) specific antibody, was utilised to assess the tumour NK cell population. Anti-NK1.1 staining was higher in all PLX4720-treated groups, ranging from 57.4 ± 6.6% to 63.0 ± 7.6, roughly twice that of any treatment without PLX4720 (Fig. 3c). NK1.1 positivity in the analysed cell population was similar in control, anti-PD-1, and anti-PD-L1-treated groups (25.5 ± 9.5 to 28.1 ± 12.3) (Supplementary Fig. 2). To understand the role of NK cells in treatment-mediated tumour shrinkage, we depleted NK cells in vivo using anti-NK1.1 antibody with PLX4720, or either combination. There was no significant increase in tumour volume despite adequate NK cell depletion, confirming NK cells are not alone sufficient to explain tumour shrinkage (data not shown).

To assess a panel of relevant cytokines, a Milliplex® multiplexed assay was performed on tumour lysate after 1 week of treatment, and again after 3 weeks (Fig. 3b). The most notable finding was a marked upregulation of IFNγ production in groups treated with 1 week of anti-PD-1/PD-L1-containing therapy, compared to control, indicating an increased Th_1_ T cell response. There was no change in IFNγ production in PLX4720-treated mice.

#### Myeloid-lineage cells: TAMs and MDSCs

**Fig. 4:**
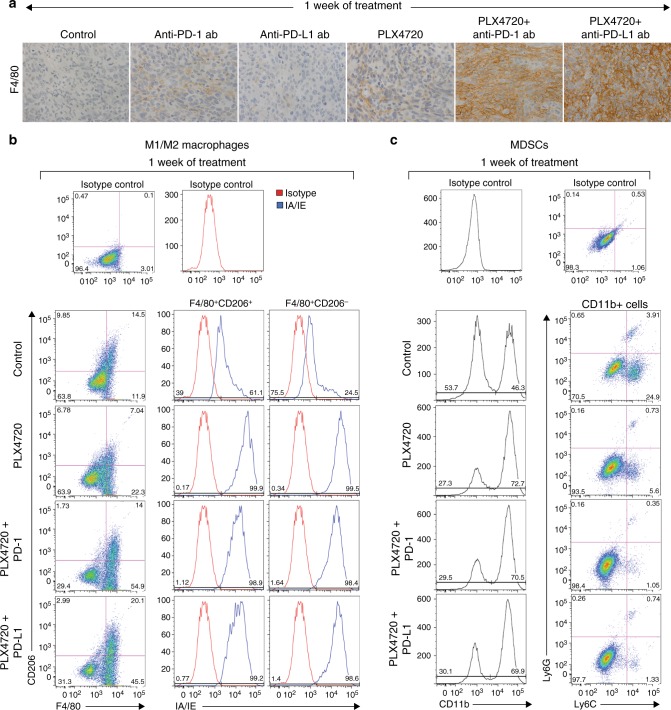
**a** One week of combination therapy led to a dramatic increase in F4/80^+^ myeloid cells. **b** All mice treated with PLX4720 singly or in combination had a dramatic increase in TAMs, >98% of which were M1-polarised (F4/80^+^CD206^−^IA/IE^+^), along with **c**. A dramatic decrease in monocytic MDSCs. *N* = 4 mice per group, repeated twice

TAMs are recognised to have a spectrum of dynamic functional polarisation; “M1-polarised” TAMs represent a pro-inflammatory population, while “M2-polarised” are typically anti-inflammatory. TAMs represent the majority of nucleated cells in some ATC tumours.^33^ Treatment with anti-PD-1 and PLX4720 monotherapies for 1 week led to a modest increase in F4/80^+^ tumour-infiltrating myeloid cells by immunohistochemistry, while treatment with either combination led to a dramatic increase in F4/80^+^ staining, compared to both control and monotherapy (Fig. 4a). Combination therapy led to a significant increase in F4/80^+^CD206^−^ cells compared to control and monotherapy, across the consistently gated cell populations—PLX4720 + anti-PD-1/PD-L1 (44.7 ± 11.3% to 46.9 ± 2.1%) vs. control, anti-PD-1, and anti-PD-L1-treated groups (15.2 ± 0.9%); more than 98% of these F4/80^+^CD206^−^ cells were IA/IE^+^, indicating an M1-polarised phenotype (Fig. 4b), a percentage that was not significantly different from control and anti-PD-1/PD-L1 monotherapy-treated groups (78 ± 36.1% to 89.1 ± 17.3%) (Supplemental Fig. 3). In contrast, F4/80^+^CD206^+^IA/IE^+^ and F4/80^+^CD206^+^IA/IE^−^ cells (M2-polarised subsets) did not significantly change, compared to control and monotherapy.

MDSCs are CD11b^+^ immature myeloid cells with immunosuppressive activity and are typically organised into early, polymorphonuclear and monocytic subsets. Since MDSCs have been implicated in ATC, we measured tumour MDSC-like cells (phenotypic markers are suggestive but not sufficient to characterise them as MDSCs) by flow cytometry.^34^ Although polymorphonuclear MDSC-like cells did not change after 1 week of treatment, we observed a significant decrease in monocytic MDSC-like cells in the PLX4720- and combination-treated groups, compared to control (Fig. 4c). Treatment with PLX4720 and combination therapy increased the proportion of CD11b^+^ cells– PLX4720 (79.2 ± 5.6%), PLX + anti-PD-1 (72.6 ± 9.8%), PLX + anti-PD-L1 (72.7 ± 10.9%) versus control (54.8 ± 7.5%). Analysis of the CD11b^+^ cells demonstrated a decrease in Ly6G^−^Ly6C^+^ (monocytic MDSC-like cells) populations in the PLX4720- (5.9 ± 3.8%), PLX + anti-PD-1- (3.4 ± 3.7%), PLX + anti-PD-L1-treated groups (4.4 ± 3.9%) versus control (17.7 ± 8.3%). No change was seen in the anti-PD-1, and anti-PD-L1-treated groups (Supplementary Fig. 4).

### Tumour regrowth occurs between 2 and 3 weeks of therapy and is associated with a decrease in the anti-tumour immune response

As mentioned earlier, tumour regrowth occurred after 1–2 weeks of continued PLX4720 monotherapy and 2–3 weeks of combination therapy during the survival experiment. To perform repeat immune profiling, a cohort of mice from the PLX4720 and combination-treated groups (*n* = 7, each) were sacrificed after 2 weeks, and another cohort of combination-treated mice (*n* = 7, each) were sacrificed after 3 weeks, just prior to meeting humane criteria for euthanisation.

#### Lymphoid-lineage cells: cytotoxic T cells, T-regulatory cells (Tregs), and NK cells

**Fig. 5:**
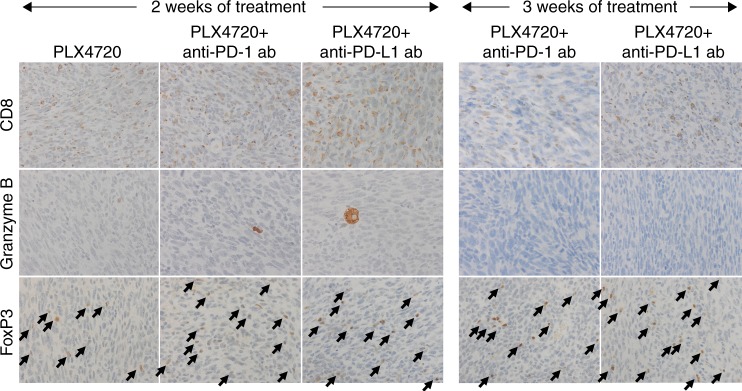
Tumour regrowth occurred after 2 weeks of PLX4720 mono- or combination therapy and coincided with **a**. A dramatic decrease in CD8^+^ cytotoxic T cells, granzyme B staining, and no change in FoxP3^+^ T cells, compared to 1 week of treatment. Three weeks of combination treatment was associated with further decreases in CD8^+^ cytotoxic T cells, granzyme B staining, and a dramatic increase in FoxP3^+^ T cells. *N* = 4 mice per group, repeated twice

CD8^+^ and Granzyme B staining steadily decreased after 2 weeks of treatment in all three groups (PLX4720, PLX4720 + anti-PD-1, PLX472 + anti-PD-L1), and more dramatically in mice treated with combination therapy for 3 weeks (Fig. 5a). Inversely, FoxP3^+^ cells increased after 2 weeks of all three treatments, and increased further after 3 weeks of combination therapy. NK cells also decreased at each timepoint for all groups (not shown). Milliplex® cytokine analysis revealed that IFNγ returned to baseline after 3 weeks, indicating a significant decline in tumour inflammation (Fig. 3b) despite continued treatment.

#### Myeloid-lineage cells: TAMs and MDSCs

**Fig. 6:**
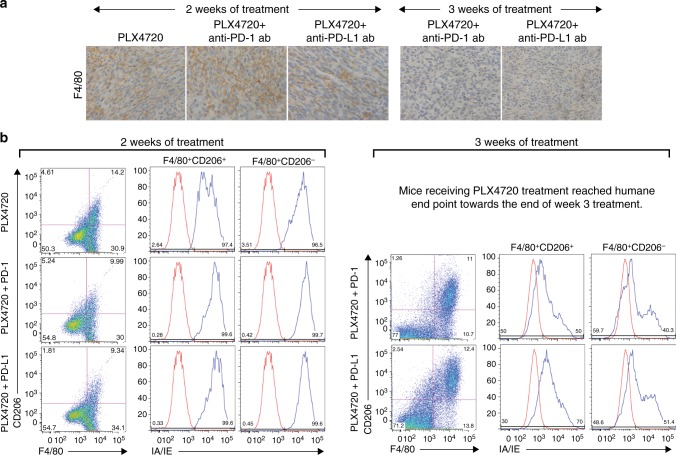
Tumour regrowth after 2 weeks of PLX4720 mono- or combination therapy coincided with **a**. An increase in F4/80^+^ myeloid cells with PLX4720, and a decrease in F4/80^+^ myeloid cells in combination-treated mice. Three weeks of combination therapy was associated with a further decrease in F4/80^+^ myeloid cells. **b** Two weeks of single or combination PLX4720 treatment did not lead to a significant change in the M1/M2 TAM populations, whereas 3 weeks of combination treatment was associated with a dramatic increase in the proportion of M2-polarised compared to M1-polarised TAMs. *N* = 4 mice per group, repeated twice

After 2 weeks of PLX4720 treatment, F4/80^+^ staining modestly increased despite the regrowth of tumour; the proportion of M1- and M2-polarised subsets did not significantly change. In contrast, F4/80^+^ staining steadily declined at week 2 in both combination treatments (Fig. 6a), and the number of F4/80^+^CD206^−^ cells decreased across the consistently gated cell populations compared to control, but there was no change in the proportion of cells with a predominantly M1-polarised phenotype (Fig. 6b). After 3 weeks of either therapy combination, F4/80^+^ staining continued to decline as tumours regrew, and F4/80^+^CD206^−^ M1-polarised cells continued to decrease. However, in contrast to week 2 where the M1- and M2-polarised cell proportions did not change, week 3 was accompanied by a dramatic decrease in the proportion of M1-polarised (F4/80^+^CD206^−^IA/IE^+^) cells from >98% to 35.4 ± 6.9% in the PLX4720 + anti-PD-1 group, and to 47.1.0 ± 3.8% in the PLX + anti-PD-L1 group. The M2-polarised (F4/80^+^CD206^+^IA/IE^−^) population dramatically rose from <2% to 54.1 ± 5.7% in the PLX4720 + anti-PD-1 group, and to 25.7 ± 6.1% in the PLX4720 + anti-PD-L1 group. There was no significant change in either polymorphonuclear or monocytic MDSC-like cells after 2 or 3 weeks in any treatment group.

## Discussion

It has become increasingly clear that a tumour’s specific genetic configuration can have a profound impact on the tumour microenvironment and anti-tumour immunity, in addition to direct effects on cell growth and proliferation.^35^ A recent GWAS showed that *BRAF*^*V600E*^-mutant thyroid cancers tend to have higher levels of T cells (both cytotoxic and regulatory T cells), M2-polarised TAMs, and lower levels of NK cells.^25,36^ Bastman et al. have demonstrated a significant CD8^+^ and Treg infiltrate in *BRAF*^*V600*^-mutant DTC and ATC.^14^ Wennerberg et al. showed that human ATC cell lines and fresh tumours were enriched in NK cells, although they had poor cytotoxic function due to low NKG2D expression. The profound clinical aggressiveness of ATC is evidence that, despite this lymphoid infiltrate, anti-tumour immunity is ineffective, likely in part due to higher PD-L1 and T cell exhaustion. Since our previous study, several groups have confirmed higher and more diffuse tumour and immune cell PD-L1 staining in a subset of patients with ATC.^14,37^ These data are particularly relevant since high PD-1 and PD-L1 expression may correlate with poorer outcomes in ATC, and PD-L1 upregulation has also been shown to be a mechanism of resistance to BRAF inhibition.^22,38^

In the current experiment, we demonstrate that the combination of PLX4720 with anti-PD-1 or anti-PD-L1 leads to a dramatic increase in mouse survival. Maximal tumour shrinkage coincided with a decreased Ki67 proliferative index, increases in tumour CD8^+^ cytotoxic T cells, FoxP3^+^ Tregs, and NK cells, as well as higher granzyme B staining and IFNγ production, confirming increased cytotoxicity. This improvement parallels findings from early phase clinical trials in melanoma that show encouraging overall response rates with a similar combination, which correlates with improved CD8^+^ T cell infiltration and IFNγ secretion.^39^ Interestingly, while the increase in NK cell number was attributable to PLX4720 alone (similar magnitude in all PLX4720-treated groups), the dramatic increase in CD8^+^ T cells and granzyme B staining appeared only with combination therapy. Although FoxP3 staining increased with PLX4720 and combination therapy, it is unclear if this represents a true increase in Tregs since FoxP3 can be transiently expressed by CD4^+^ and CD8^+^ T cell populations during activation or rescue from exhaustion.^40^

Unfortunately, despite the profound reduction in tumour volume, doubling of survival time, and favourable immune infiltrate during maximal response to treatment, no complete responses were seen and tumour regrowth emerged after 2–3 weeks of combination therapy, quickly leading to mouse death. Tumour regrowth was associated with decreases in CD8^+^ T cells, NK cells, and loss of granzyme B and IFNγ production, confirming dampened inflammation. While these changes are important, it is now clear that the anti- or pro-tumourigenic nature of the lymphoid infiltrate is not only dependent on the specific type and ratio of various lymphocytes (CD4^+^ “helper” T cells, CD8^+^ cytotoxic T cells, cytotoxic NK cells, regulatory T cells), but also their location within the tumour, and the context of the surrounding cellular (e.g. myeloid cells, dendritic cells) and the cytokine milieu. These observations underscore the importance of thoroughly assessing the immune microenvironment using in vivo models with a full immune cell repertoire.

Indeed, maximal response to combination therapy was also associated with significant changes in the myeloid-lineage compartment, including an increase in predominantly M1-polarised TAMs. TAMs arise from differentiation of circulating MDSCs and monocytes, as well as from recruitment of tissue macrophages. TAM polarisation is heavily influenced by the surrounding immune milieu of B-cells, T_h_2 T cells, fibroblasts, granulocytes, and tumour cells. While M1-polarised TAMs facilitate inflammation, M2-polarised TAMs hamper adaptive immunity, and facilitate invasion, metastasis, and the epithelial-to-mesenchymal transition, among other mechanisms. TAMs dampen immunity through production of immunosuppressive cytokines (e.g. IL-10 and TGFβ) and prostacyclins, depletion of metabolic substrate, and T cell/NK cell inhibition.^41^ In thyroid cancer, increased TAM infiltration is found with increasing de-differentiation in PDTC and ATC.^33,42^ Although lymphocytes have been the primary target in mechanistic studies of anti-PD pathway efficacy, PD-1 expression has recently been recognised on TAMs, and stimulation leads to inhibited phagocytosis.^43^ In light of these findings, it is not surprising that the emergence of resistance to combination therapy in our study was marked by a decrease in the number of TAMs with a dramatic increase in the ratio of M2:M1-polarised cells; it is likely that this change, at least in part, is responsible for the reduction in both T and NK cell number and function. Both TAMs and MDSCs have been implicated not only in tumour development, but in resistance to kinase and checkpoint inhibitors in other tumour types.^35,44,34,45,46^ Therapies that target TAM recruitment, proliferation and polarisation could be a promising component of future combination therapies.^41^

MDSCs are a heterogenous population of circulating immature myeloid cells that arise from dysregulated myelopoiesis in the setting of chronic inflammation, uncontrolled cell growth, trauma, etc. Major subtypes include early (eMDSC), polymorphonuclear (PMN-MDSC), and monocytic (M-MDSC) MDSCs, and although they differ in their phenotype and potency, they share a common ability to suppress effector T cell activity, similar to M2-polarised TAMs.^44^ MDSCs have been detected in the peripheral blood of patients with DTC and ATC, and appear to correlate with disease stage.^45^ At the time of maximal response to combination therapy, mouse tumours demonstrated a significant decrease in the monocytic MDSC-like cell fraction. Despite this significant decrease, MDSC-like cells remained low during tumour regrowth, suggesting that MDSCs may play a role in response to, but not in resistance to combination therapy.

Our study has several limitations, many of which have been reviewed in our previous manuscripts.^24,26^ It is unclear if the genetically engineered TBP-3743 ATC cell line is comparable to human patients with ATC, especially given the relationship between genetic and epigenetic changes and perturbations in host immunity, but the transgenic model from which TBP-3743 was derived has been shown to recapitulate the dedifferentiated histology, temporal aggressiveness, and gene expression profile of human ATC. Despite this limitation, our model is relentlessly aggressive, which is unfortunately a feature shared by many patients with ATC; down titration of injected cell number has failed to significantly alter the course of disease. Since orthotopic transplantation of TBP-3743 into syngeneic mice is the only validated *Braf*^*V600E*^-mutant immunocompetent model of ATC, we are actively working to develop immunocompetent orthotopic mouse models with alternative ATC cell lines.

Given the focus of this study on establishing the effectiveness and preliminary mechanism of combination therapy, along with the rapid lethality of our animal model, we chose a concurrent treatment algorithm. It is possible that other treatment algorithms, such as sequential therapy, may be better tolerated and more clinically effective, due to capitalisation on immune cell recruitment through MAPK inhibition; this approach is under investigation in melanoma and will be an area of future study.^39^ Although the addition of MEKi to BRAFi has recently been approved by the FDA in human ATC, we chose to focus on the combination of BRAFi with anti-PD-1/PD-L1 given the critical role of MEK signalling in the activation of CD8^+^ and CD4^+^ T lymphocytes, and thus the theoretical risk that any gains from MEK inhibition might be abrogated by simultaneous negative effects on T cells. However, triple therapy with BRAFi, MEKi, and immunotherapy is a promising future direction since immunotherapy may counteract the negative immune effects of MEKi.^47^ Finally, although we identified M2-polarised TAMs and MDSCs using their phenotypic markers, phenotypic assessment is necessary but not sufficient for complete characterisation; demonstration of immunosuppressive function is required to confirm their presence and significance. The use of depletion experiments for each immune cell will confirm the relative importance of each during maximal response and tumour regrowth. We aim to address these limitations in ongoing experiments.

In summary, we demonstrate that the combination of BRAFi and anti-PD pathway immunotherapy dramatically reduces tumour volume and improves survival in an immunocompetent murine model of orthotopic ATC, and that maximal treatment response and regrowth of tumour correlate with significant changes in the tumour immune cell infiltrate. Combination therapy is a promising modality in ATC and other cancers to maximise treatment efficacy, boost response rates, minimise toxicity, and forestall the development of resistance. Multiple clinical trials utilising this and similar treatment combinations are already underway, and it is imperative to rapidly disseminate this preclinical information to clinicians. Additionally, ongoing work to identify predictors and mechanisms of response and resistance is critical to identify optimal candidates for specific therapies, and to further inform the rational addition of “targeted” agents. Immune cell profiling is an auspicious modality to complement mutational assessment in the context of furthering the goal to deliver truly “personalised” medicine.

## Electronic supplementary material

Supplemental Figures

## Data Availability

The datasets generated and/or analysed during the current study are available from the corresponding author on reasonable request.
