# Supplementary material for: Combinations of BRAF inhibitor and anti-PD-1/PD-L1 antibody improve survival and tumour immunity in an immunocompetent model of orthotopic murine anaplastic thyroid cancer
Source: Br J Cancer. 2018 Oct 17;119(10):1223–32. doi: 10.1038/s41416-018-0296-2 (PMC6251038; doi:10.1038/s41416-018-0296-2)
Supplement: Supplementary file 1 — Supplemental Figures [file 41416_2018_296_MOESM1_ESM.doc]

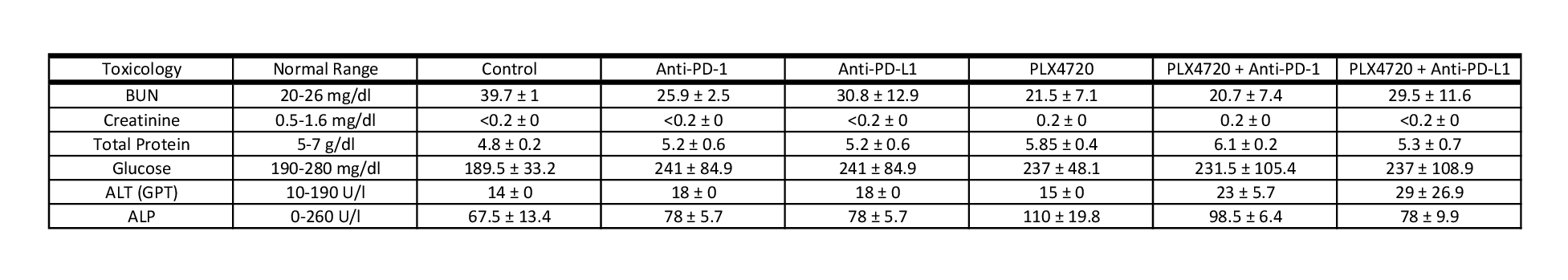
**Supplementary Fig. 1**

Toxicology assessment of mouse serum from each group after 2 weeks of treatment. Elevated BUN and decreased creatinine correlated with physical deconditioning.


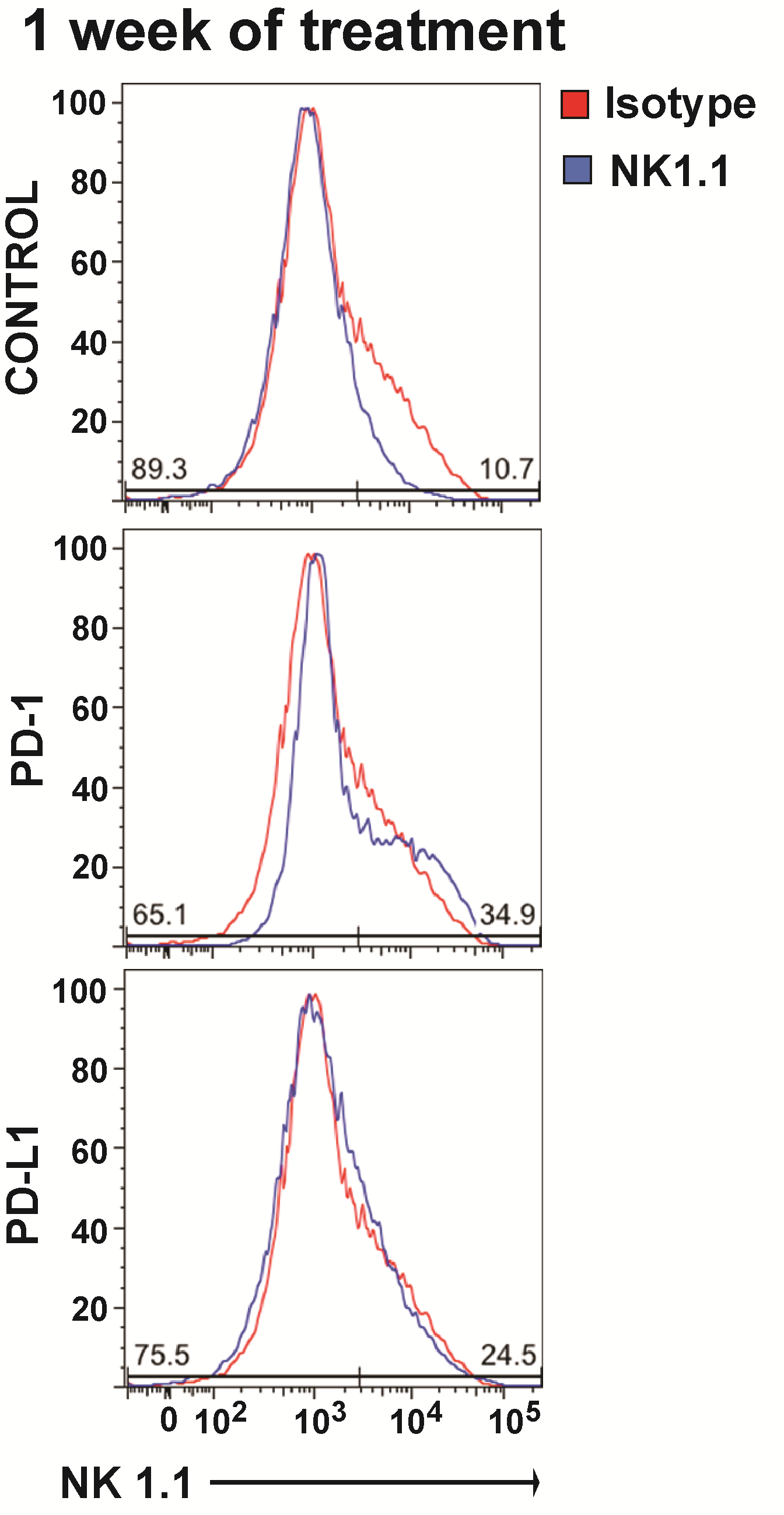


**Supplementary Fig. 2**

NK-cells (representative samples shown using NK1.1 antibody) did not significantly change after one week of treatment with anti-PD-1 or anti-PD-L1 monotherapies.


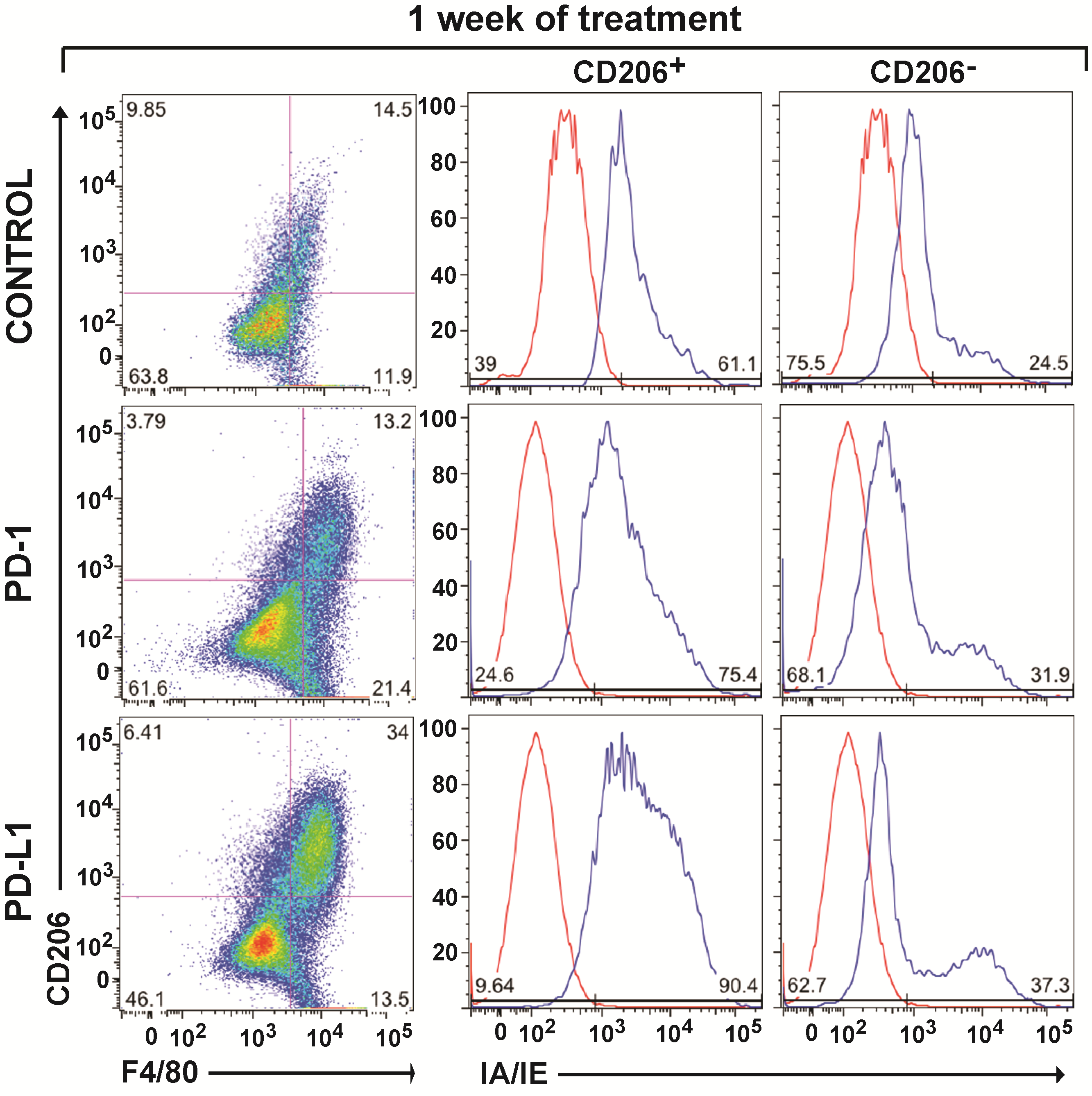


**Supplementary Fig. 3**

One week of treatment with anti-PD-1 led to a modest increase in M1-polarized TAMs while anti-PD-L1 led to minimal changes compared to the dramatic changes with combination therapy.


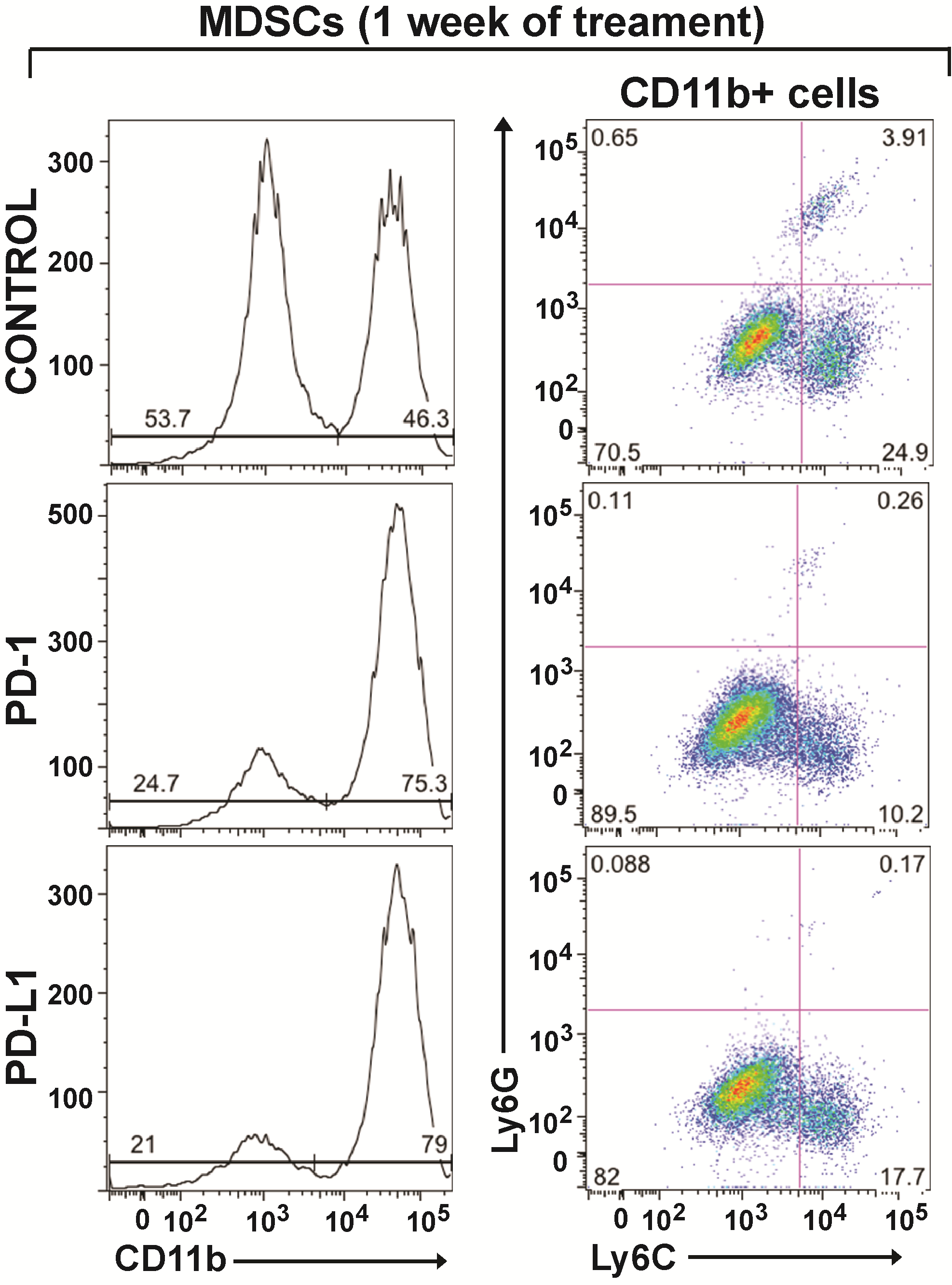


**Supplementary Fig. 4**

One week of treatment with anti-PD-1 or anti-PD-L1 did not significantly change the polymorphonuclear or monocytic MDSC populations as was seen in combination therapy.
